# Calcium Fluxes in Work-Related Muscle Disorder: Implications from a Rat Model

**DOI:** 10.1155/2019/5040818

**Published:** 2019-09-30

**Authors:** J. Hadrevi, M. F. Barbe, N. Ørtenblad, U. Frandsen, E. Boyle, S. Lazar, G. Sjøgaard, K. Søgaard

**Affiliations:** ^1^Department of Public Health and Clinical Medicines, Occupational and Environmental Medicine, Umeå University, 901 87 Umeå, Sweden; ^2^Department of Anatomy and Cell Biology, Lewis Katz School of Medicine of Temple University, 3500 North Broad St., Philadelphia, PV 19140, USA; ^3^Department of Sports Science and Clinical Biomechanics, Faculty of Health Sciences, University of Southern Denmark, Odense, Denmark

## Abstract

**Introduction:**

Ca^2+^ regulatory excitation-contraction coupling properties are key topics of interest in the development of work-related muscle myalgia and may constitute an underlying cause of muscle pain and loss of force generating capacity.

**Method:**

A well-established rat model of high repetition high force (HRHF) work was used to investigate if such exposure leads to an increase in cytosolic Ca^2+^ concentration ([Ca^2+^]_i_) and changes in sarcoplasmic reticulum (SR) vesicle Ca^2+^ uptake and release rates.

**Result:**

Six weeks exposure of rats to HRHF increased indicators of fatigue, pain behaviors, and [Ca^2+^]_i_, the latter implied by around 50–100% increases in pCam, as well as in the Ca^2+^ handling proteins RyR1 and Casq1 accompanied by an ∼10% increased SR Ca^2+^ uptake rate in extensor and flexor muscles compared to those of control rats. This demonstrated a work-related altered myocellular Ca^2+^ regulation, SR Ca^2+^ handling, and SR protein expression.

**Discussion:**

These disturbances may mirror intracellular changes in early stages of human work-related myalgic muscle. Increased uptake of Ca^2+^ into the SR may reflect an early adaptation to avoid a sustained detrimental increase in [Ca^2+^]_i_ similar to the previous findings of deteriorated Ca^2+^ regulation and impaired function in fatigued human muscle.

## 1. Introduction

Work-related muscle pain is considered a public health problem and increases the incidence of sick leave absences in otherwise healthy individuals [1]. Repetitive work tasks are known risk factors for causing work-related musculoskeletal disorders and chronic muscle pain [2–4]. The muscular pathogenic symptoms of work-related muscle pain include stiffness, weakness, and increased tension [5]. The process of controlling the production of force within the muscle, known as excitation-contraction-relaxation coupling, requires a tight regulation of the intracellular cytosolic-free Ca^2+^ concentration ([Ca^2+^]_i_) in muscles enabling the activation of the contractile apparatus, while protecting the cell from deleterious [Ca^2+^]_i_ overload. This is permitted through an instantaneous release of large amounts of Ca^2+^ through the sarcoplasmic reticulum (SR) Ca^2+^ release channel and ryanodine receptor (RyR), thereby increasing [Ca^2+^]_i_ and a subsequent, almost simultaneous, reuptake of Ca^2+^ into the SR by the SR ATPases (SERCA) together with buffering of Ca^2+^ inside the SR by the protein calcequestrin (Casq1) [6–8].

Repetitive or sustained contraction of skeletal muscle can lead to a progressive loss in the ability to produce the desired force, known as muscle fatigue, and has been linked to impaired Ca^2+^ regulation and SR Ca^2+^ release rates [9, 10]. Ultimately, excessive repetitive muscle contractions can cause prolonged force depression and muscle cell damage. While muscle fatigue relates to impaired SR Ca^2+^ release rate [11, 12], cell damage relates to a sustained increase in [Ca^2+^]_i_ above normal for a prolonged time period [13]. Disruption of homeostasis can cause Ca^2+^ overload due to Ca^2+^ leakage from the SR, impaired Ca^2+^ uptake into the SR, and/or increased cell membrane Ca^2+^ influx [14, 15]. These disturbances may activate intracellular Ca^2+^-dependent proteins such as calpains that degrade intracellular proteins, cellular membranes, and nuclear DNA [16].

In line with this, dysfunctional Ca^2+^ homeostasis has been shown in skeletal muscles of patients suffering from chronic neck shoulder pain [17], and a study in myagic muscle [18] showed a decreased abundance of Casq1 together with an increased abundance of SERCA. These findings may indicate an increased uptake of Ca^2+^ into the SR yet reduced the buffering capacity within the SR. Ca^2+^ regulatory excitation-contraction coupling properties are key topics of interest in the development of work-related muscle myalgia and may constitute an underlying cause of weakness and reduced capacity to rapidly produce force and muscle pain [19–22]. Therefore, these associations between Ca^2+^ and pain intrigued us to further examine the intramuscular excitation-contraction coupling properties during development of work-related muscle pain.

Since animal models may be more apt than human studies for highly invasive procedures to analyze subcellular mechanisms, we utilized a well-established rat model of high repetition high force (HRHF) work compared with food-restricted control (FRC) rats to investigate if declines in sensorimotor behaviors were related to potential changes in intracellular Ca^2+^ homeostasis and injury markers. The rat model has shown grip strength declines in parallel with pain-related symptoms in rats [23–25], similar to findings in humans [24]. Here, the rat model was used to investigate subcellular responses in intramuscular Ca^2+^ fluxes to further elucidate the underlying mechanisms of work-related musculoskeletal disorders in workers. We hypothesized an increase in [Ca^2+^]_i_ in HRHF rat muscles, compared to FRC rats, and adaptive compensatory changes in SR Ca^2+^ uptake and release rates. Since [Ca^2+^]_i_ cannot be measured directly in an in vivo setting, a number of crucial and well-investigated proteins were chosen to elucidate possible Ca^2+^ flux rates, pCalmodulin kinase (pCam), a protein indicative of [Ca^2+^]_i_; RyR, a protein related to SR Ca^2+^ release; SERCA1, a protein regulating SR Ca^2+^ uptake; and finally Casq1, a protein buffering Ca^2+^ inside the SR. As pain is a perception and always self-reported, an animal model can only observe and test motor and sensory behaviors as indications of fatigue and pain. We analyzed for voluntary motor behaviors suggestive of fatigue and sensory behaviors suggestive of pain or discomfort, as well as inflammatory cytokines and Hsp72 as injury markers [26, 27]. All original data from behavioral and tissue analyses used to support the findings of this study are available from the corresponding author upon request.

## 2. Methods

### 2.1. Animals

Experiments were approved by the Institutional Animal Care and Use Committee of Temple University (Animal Protocol number 4476) and were in compliance with NIH guidelines for the humane care and use of laboratory animals. All rats were housed in an animal facility in separate cages with a 12-hour light-dark cycle, free access to water, and environmental enrichment daily in their home cages (chew toys and tunnels). Studies were conducted on young adult (3.5 months of age at the onset of experiments), female, Sprague-Dawley rats. As estrogen and sex influence the exercise-related disturbances of Ca^2+^ homeostasis involved in subsequent muscle damage [28–30], only female Sprague-Dawley rats were used since human females have a higher incidence of work-related musculoskeletal disorders than males [31–33]. All rats were food-restricted to motivate interest in food reward pellets to no less than 5% lower than age-matched normal controls (used for weight-matching purposes only). For this, all rats were weighed at least weekly throughout the experiment and food adjusted accordingly. In addition to 480 food pellet rewards provided three days per week, all rats received Purina rat chow daily (approximately 5 grams per day per 100 grams of body weight) and were allowed to gain in weight over time, as shown in Supplementary [Supplementary-material supplementary-material-1]. The control rats received daily allotments of food pellets and rat chow at matched levels as HRHF rats.

Twelve rats were included in the present study. Six rats were allocated randomly to perform the HRHF operant reaching and grasping task for six weeks. The remaining six rats served as food restriction controls (FRCs) and did not perform the task. An overview of the behavioral and tissue analyses performed is presented in Table 1.

### 2.2. Behavioral Apparatuses

**Figure 1:**
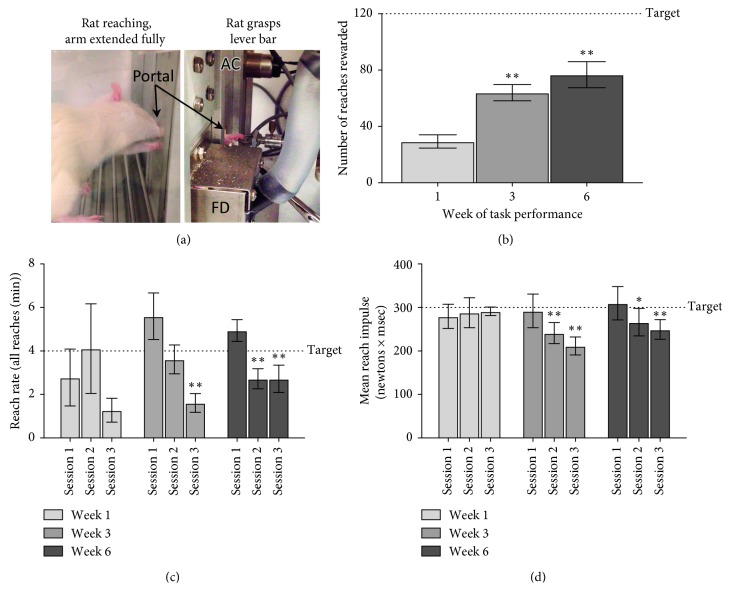
Task performance after 1, 3, and 6 weeks of repetitive reaching and grasping at high repetition high force (HRHF) levels. (a) Rat is shown reaching through a portal to pull on a lever bar attached to a force transducer for a food reward (AC = auditory clicker for cueing; FD = food dispenser). (b) HRHF rats had progressively increasing numbers of reaches rewarded, although they remained below the target of 120 rewards/session in each week, compared to week 1; ^*∗∗*^*p* < 0.01, compared to week 1. (c) HRHF rats showed reduced reach rates below the target of 4 reaches/minute in session 3 of week 3 and in sessions 2 and 3 of week 6, compared to session 1 of the same week. (d) HRHF rats show a reduced ability to maintain target reach impulse levels in sessions 2 and 3, of weeks 3 and 6, compared to session 1 of the same week. In (c) and (d), ^*∗*^ and ^*∗∗*^ represent *p* < 0.05 and *p* < 0.01, compared to session 1; mean ± SEM shown.

Sixteen custom-designed behavioral apparatuses (Custom Medical Research Equipment, Glendora, NJ) were integrated into standard open field boxes (Med Associates, St. Albans, VT), as previously described in detail [34] and as depicted in Figure 1(a).

### 2.3. Procedure for Repetitive Reaching and Grasping Task

HRHF rats were first exposed to a six-week learning period, termed “shaping” to learn the reaching and handle-pulling task for 15 min/day, 5 days/wk [34]. They ramped upwards from naïve towards a high force grasping task with a required pull on the lever bar of 1.08 to 1.27 N (41 to 48% of their maximum pulling force) for a food reward [35, 36].

The “shaped” rats then went on to perform the HRHF task simulating occupational repetitive work for 1.5 hours/day, 3 days/week, for an additional six weeks. The daily task was divided into three 30-minute sessions separated by 1.5 hours each to avoid satiation. The rats had to grasp the force lever bar and exert an isometric pull at a target grasp time of 200 milliseconds and the required target force. The preferred reach limb (RL) of HRHF task rat used to grasp and reach the lever bar was recorded during each session. The contralateral limb was often used as a support limb (SL) against the wall of the chamber as shown in Figure 1(a) and as reported previously [37].

### 2.4. Determination of Reach Performance Behaviors in HRHF Rats

Force lever data were recorded continuously during each task session for later calculation of dependent variables (number of reaches rewarded, reach rate (i.e., all reaches/minute), grasp force, and grasp time) via an executable automated script (MatLab; Mathworks, Natick, MA) as described previously [38, 39]. Grasp time was defined as the mean average time (in msec) spent on all recordable reaches. The mean reach impulse mean grasp force (N) *×* mean grasp time (msec) was calculated. Data for each variable were calculated on the last day of weeks 1, 3, and 6. Week 1 was used as the baseline to reach performance variables.

### 2.5. Other Sensorimotor Behavioral Tests

Several sensorimotor assays were assayed in both HRHF and FRC rats (Table 1). Maximum reflexive grip strength, forelimb agility (the forehead sticker removal test), forepaw mechanical sensitivity, and spontaneous behavioral changes were studied as indicators of pain or discomfort [40–43]. These behavioral tests were performed at the naïve time-point, after food restriction (FR, 6 weeks), after the initial shaping period (which was HRHF week 0), and every three weeks thereafter. The person carrying out these tests was blinded to group assignment. Behavioral procedures were conducted at the same time per day to minimize diurnal-related factors. Reflexive grip strength of the forelimbs was measured, bilaterally, using a grip strength meter as previously described [44]. This is a “break” test in which rats are pulled backwards until their hold on a bar attached to a force transducer system is broken (i.e., reflexive). Maximum reflexive grip strength is reported for each limb individually out of 3–5 tests/testing period. A forehead sticker removal test was used to determine functional agility and discomfort of each forelimb, as previously described [43]. Briefly, forelimb movements were scored as follows: 0 = no attempt to remove the sticker and 5 = successful removal of the sticker. Forepaw mechanical sensitivity was assessed, bilaterally, using 0.4 von Frey filaments, as previously described [25]. Data were reported RL and SL of HRHF rats (termed, respectively, hereafter as HRHF-RL and HRHF-SL) and for the right and left limbs of FRC rats (termed hereafter as FRC-R and FRC-L). Lastly, trained observers tracked changes in spontaneous behaviors indicative of discomfort occurring during each period of HRHF task performance. Bilateral pulling of the lever bar, supinated pull, and guarding were recorded upon occurrence. Data from the last day of each task week are reported for HRHF rats.

### 2.6. Tissue Collection

In task week six, rats were euthanized 36 hours after completion of the final task session (to avoid acute effects of muscle activity) using sodium pentobarbital (120 mg/kg body weight) for collection and assay of inflammatory and metabolic changes in the forelimb muscles. The following tissues were collected: flexor digitorum, extensor digitorum, trapezius, and supraspinatus muscles (termed Flex, Ext, Trap, and Supra, respectively). These tissues were collected bilaterally from all rats, i.e., HRHF-RL, HRHF-SL, FRC-R, and FRC-L. Each muscle was further divided into distal and middle regions (termed region A and B, respectively) and homogenized separately. The distal region of the Trap muscle was the region attached laterally on the scapula spine, while the middle region was located more superiorly towards the head and cervical vertebrae. Following collection, muscle samples were blotted on a filter paper and placed on ice. Each muscle region was weighed and dissected into pieces of at least 25 mg and treated further as described below.

### 2.7. ELISA

Portions of distal and middle regions (A and B) were combined for each Flex muscle from HRHF-RL, HRHF-SL, FRC-R, and FRC-L limbs and then stored individually at −80°C until being homogenized using previously described methods [45]. Supernatants were analyzed using commercially available single plex ELISA kits for IL-1alpha, IL-1beta, TNFalpha, and IL-10, as previously described [45]. These muscle homogenates were also assayed for the inducible isoform of heat shock protein 72 (Hsp72) levels using a single-plex ELISA kit (Enzo Life Sciences, Inc, Farmingdale, NY), using the manufacturer's protocol. Each sample was run in duplicate. ELISA results were normalized to total protein concentration as measured by the BCA assay (Pierce Biotechnology, Rockford, IL) and presented as picograms of protein per micrograms of total protein.

### 2.8. SR Vesicle Ca^2+^ Release and Uptake Rates

SR Ca^2+^ release and uptake rates were measured in SR vesicles from all muscles of the HRHF and FRC. Portions of each muscle region were homogenized (1 : 20 weight : volume) in ice-cold buffer of 0.3 M sucrose, 1 mM EDTA, 10 mM NaN_3_, 40 mM Tris-base, 40 nM L-Histidine, and H_2_O (pH 7.8) added at a concentration of 1 : 20 (weight : volume). Tissues were homogenized with a 5 mm generator (∼16,000 rpm, level 4) in three, 15-second bursts, separated by 15-second pauses between each burst (with tissue kept on ice during homogenization). Tissues were transferred to Eppendorf tubes, flash frozen in liquid N_2_, and stored at −80°C until analyzed.

Homogenates were analyzed for SR vesicle Ca^2+^ release and uptake rates, as previously described [11, 46]. Muscle homogenates (70 *μ*l) were mixed with 2 ml assay buffer (165 mM KCl, 22 mM HEPES, 7.5 mM oxalate, 11 mM NaN_3_, 5.5 *μ*M N,N,N′,N′-tetrakis(2-pyridylmethyl)ethylenediamine (TPEN), 20 *μ*M CaCl_2_, and 2 mM MgCl_2_ (pH 7.0 at 37°C), and the reaction initiated by adding ATP to a final concentration of 5 mM [Ca^2+^] was determined fluorometrically (20 Hz, Ratiomaster RCM, Photon Technology International, Brunswick, NJ, USA) using the fluorescent Ca^2+^ indicator indo-1 (1 *μ*M). When [Ca^2+^] reached a plateau (Nadir Ca^2+^), SR Ca^2+^ uptake was blocked by adding Cyclopiazonic acid (40 *μ*M) and Ca^2+^ release was initiated by adding 4-chloro-m-cresol (5 mM), and the fluorescence followed for at least 30 seconds. All raw data for Ca^2+^ release and uptake were imported into Matlab version 7.0.1 (the MathWorks, Natick, MA) and mathematically analyzed (Curve Fitting Toolbox ver. 1.1.1; the MathWorks) [46]. Curve fitting of Ca^2+^ uptake was performed with data points between a free [Ca^2+^] of 800 nM and the free [Ca^2+^] 20 seconds prior to initiating Ca^2+^ release (*r*^2^ > 0.99 for all data sets). The time for the free [Ca^2+^] to decrease by 63% of the initial free [Ca^2+^] (*τ*) was calculated as 1/*b* from the equation, *y* = *ae*^−*bt*^ + *c*, where *y* is the free [Ca^2+^], *t* is time and *a*, and *b* and *c* are constants assigned from Matlab. The onset of Ca^2+^ release was defined as the time when [Ca^2+^] increased above nadir [Ca^2+^]. The data points during the first 30 seconds of release were mathematically fitted to the equation, *y* = *a* [1 − *e*^−*b* (t−c)^]. The values obtained for SR Ca^2+^ release and uptake rates are expressed as arbitrary units of Ca^2+^ min^−1^ g^−1^ protein and the inverse rate constant *τ*, in seconds, respectively. Assays of uptake and release rates of Ca^2+^ were performed in triplicates (a few in duplicates due to limited tissue homogenate). Protein content in the muscle homogenate was measured in triplicates using a standard kit (Pierce BCA protein reagent no. 23225).

### 2.9. Western Blot Analyses

Western blot analyses for pCam, RyR1, Casq1, and SERCA1 protein expressions were performed on tissue lysates also used for the analysis of SR vesicle Ca^2+^ uptake and release rates on region B samples from HRHF-RL and FRC-R muscles. Protein content in the tissue lysate was measured in triplicate using a standard kit (Pierce BCA protein reagent no. 23225, Pierce Inc). Laemmli buffer was added to lysates, the samples were heated at 90°C for three minutes, shortly vortexed, and spun in a microcentrifuge, and equal amounts (20 mg in 10 *μ*l) were separated by SDS-PAGE (Miniprotean TGX, BioRad, Hercules, CA, USA) using precast 4–12% BIS TRIS NuPAGE gels (Thermo Fischer, Denmark) at 100 V for 55 minutes. Gels were blotted (Transblot cell, Bio-Rad, 250 mA, 1 h) onto polyvinylidene difluoride membranes (Immun-Blot PVDF 162-0177, BioRad, Hercules, Ca, USA) blocked for 30 minutes in 5% milk dissolved in TBS/0.05% Tween-20 (TBST) and incubated overnight at 4°C with primary antibodies diluted 1 : 1000 in 4% BSA in TBST with shaking: mouse anti-phospho-CaMKII (Thr286) (pCam, MAI-047, Thermo Scientific); mouse anti-ryanodine receptor 1 (RyR1, ab2868, Abcam); rabbit anti-calsequestrin-1 (Casq1, C0618, Sigma-Aldrich); mouse anti-SERCA1 ATPase (SERCA1, ab2819, VE121G9, Abcam); and rabbit anti-GAPDH (GAPDH, 14C10 #2118, Cell Signaling). Membranes were washed in TBS/0.05% Tween (28358, TS/170-6531, BioRad, Hercules, Ca, USA) and then incubated for one hour with appropriate HRP-conjugated secondary antibodies diluted 1 : 3000. Membranes were visualized with Immun-star western kit (#170-5070, BioRad, Hercules, CA, USA). To conserve samples, prior to immunostaining as described above, membranes were cut into upper and lower halves and one-half stained for GAPDH (the loading control) and the other for the protein of interest, as appropriate for the molecular weight of the protein of interest. For example, the upper half of the membrane probed for Casq1 (∼60 kDa) was stripped and reprobed for SERCA (110 kDa). Images were obtained using ChemiDoc XRS (Bio-Rad Laboratories, Inc.) and subsequently analyzed for densitometry using Bio-Rad image lab software. All band densities were normalized to internal control samples loaded onto each gel (specifically, equal aliquot of the same control animal was loaded onto each gel). Normalized bands were compared to GAPDH, and these ratios were graphed for representative blots. Gels and blots were repeated until four to five different samples per group were assayed, for a minimum of 5 gels per protein of interest.

### 2.10. Statistical Analysis

Data are reported as mean and SEM, GraphPad PRISM v.7.0. One-way repeated measures ANOVAs were used to examine the number of reaches rewarded. Two-way repeated measures ANOVAs were used to analyze the reach rate and mean reach impulse across the three sessions, using the factors session and week. Grip strength, forelimb agility, and forepaw mechanical sensitivity results were assayed using two-way repeated measures ANOVAs, using the factors week and group, with the group data separated by limb (HRHF-RL, HRHF-SL, FRC-R and FRC-L). Spontaneous behavioral changes suggestive of discomfort tracked during task performance were compared using nonparametric Kruskal–Wallis tests, with data compared to week 1 HRHF results. ELISA cytokine results were analyzed using two-way ANOVAs and the factors group (HRHF and FRC) and limb (RL/R and SL/L) for the Flex muscles. Linear mixed models were conducted in Stata version 15.0 to determine if SR Ca^2+^ vesicle uptake and release differed by group (HRHF versus FRC), location within the muscle (region A versus B), or muscle (Flex, Ext, Supra, and Trap). Western blot results from the preferred reach limb muscles were assayed using two-way ANOVAs and the factors group (HRHF and FRC) and muscle. ANOVAs were followed by Tukey post hoc tests for multiple comparisons. Adjusted *p* values for the Tukey post hoc results of <0.05 were considered significant for all comparisons.

## 3. Results

### 3.1. Increased Indication of Fatigue with HRHF Task

We observed that HRHF rats had significant differences in the number of reaches rewarded (ANOVA *p*=0.008), which progressively increased across weeks of task performance (*p*=0.01 each in weeks 2 and 3, compared to week 1; Figure 1(b)), although the HRHF rats never met the target of 120 food pellets rewarded per day in any week. There were significant differences in the mean reach rate by session (*p*=0.0008; Figure 1(c)), with post hoc analyses revealing lower reach rates in session three of week 3 and sessions two and three of week 6, compared to session one of the same week (*p* < 0.01 each). There were also significant differences in mean reach impulse (grasp force × grasp time) by week (*p* < 0.0001) and session (*p*=0.0001; Figure 1(d)). Post hoc analyses revealed lower mean reach impulses in sessions two and three in both task weeks of 3 and 6, compared to session one in these same weeks (*p* < 0.05 each).

### 3.2. Increased Indication of Muscle Discomfort

**Figure 2:**
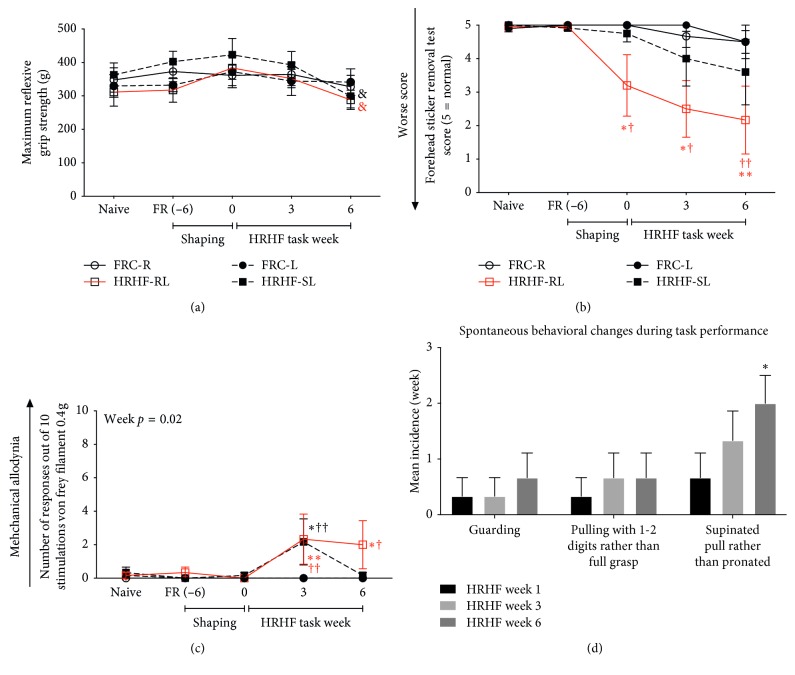
Sensorimotor behavioral test results across weeks. (a) Maximum reflexive grip strength showed decreased grip strength in both the HRHF rat reach limbs (HRHF-RL) and HRHF-SL in week 6, compared to week 0 levels. (b) Forelimb agility/discomfort was tested using a forehead sticker removal test and showed a reduced ability (or reduced willingness to participate) in HRHF-RL in task week 0 through 6, compared to naïve and food restricted control (FRC)-R limbs. (c) Forepaw mechanical sensitivity was tested using 0.4 g von Frey filaments and revealed increased numbers of forepaw withdrawal responses (indicative of increased sensitivity) in week 3 after stimulation with the filament in HRHF-RL and HRHF-SL, compared to FRC-R and FRC-L, respectively, and an increase in week 6 in HRHF-RL, compared to FRC-R. (d) The HRHF rats showed higher incidence of spontaneous behaviors indicative of discomfort in task week 6 than week 1. ^*∗*^ and ^*∗∗*^*p* < 0.05 and *p* < 0.01, compared to week 1; ^†^ and ^††^*p* < 0.05 and *p* < 0.01, compared to age-matched FRC rats; *p* < 0.05, compared to week 0; mean ± SEM shown.

Maximum reflexive grip strength showed a significant difference by week (*p*=0.006). Post hoc tests revealed that grip strength had declined in both the HRHF-RL and HRLF-SL, compared to week 0 levels (*p* < 0.05 each; Figure 2(a)). The forelimb agility/discomfort scores showed significant differences by week (*p*=0.003) and group (*p*=0.0005; Figure 2(b)), with post hoc tests showing significant declines in forelimb agility (or reduced willingness to participate in the assay) in the HRHF-RL (shown in red) in weeks 0, 3, and 6, compared to naïve (*p* < 0.05 or  < 0.01). Forepaw mechanical withdrawal responses showed a significant difference by week for the 0.4 g von Frey filament (*p*=0.02, Figure 2(c)), with post hoc tests showing increased withdrawals to the 0.4 gram filament in week 3 in both HRHF forepaws, compared to FRC rats (*p* < 0.05 each) and to the 0.4 g filament in week 6 in HRHF-RL forepaws, compared to FRC-R rats (*p* < 0.05; Figure 2(b)). Tracking of spontaneous behaviors suggestive of muscle discomfort during task performance showed a significant increase in supinated pulling of the lever bar rather than the typical pronated pull in earlier weeks (Figure 2(d)).

### 3.3. Muscle Inflammatory Cytokine and Heat Shock Protein Levels

**Figure 3:**
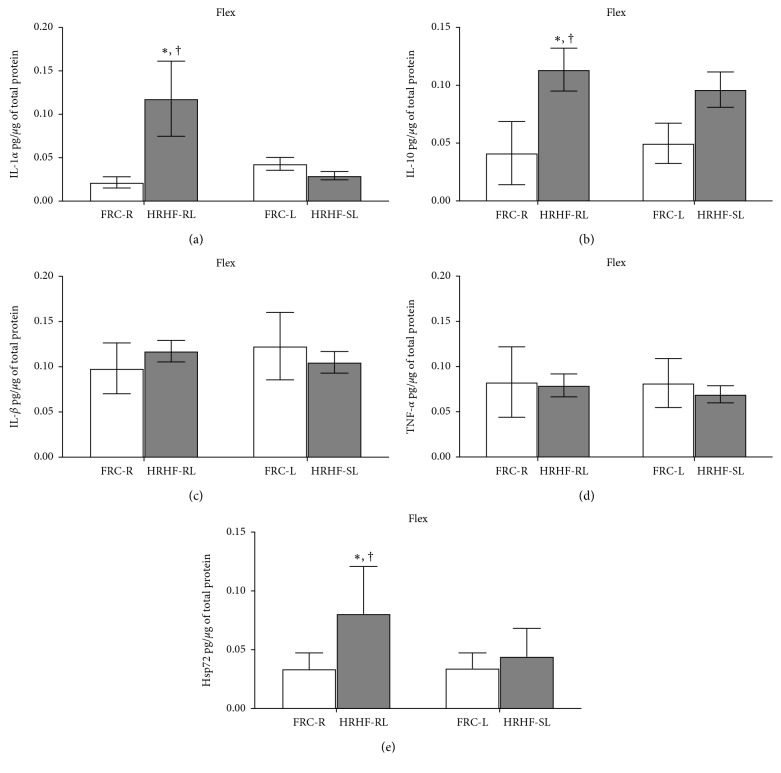
Levels of IL-1alpha, IL-10, IL-1beta, TNFalpha, and heat shock protein 72 (Hsp70) in flexor digitorum (Flex) muscles, tested using ELISA. Portions of regions A and B (see Methods) were combined for each analysis. (a, b) IL-1alpha and IL-10 levels in Flex muscles were significantly increased in HRHF-RL, compared to FRC-R and HRHF-SL. (c, d) IL-1beta and TNFalpha levels in the Flex muscles did not differ between the groups. (e) Hsp72 levels were significantly increased in HRHF-RL, compared to HRHF-SL and FRC-R limbs. ^*∗*^*p* < 0.05, compared to FRC-R; ^†^*p* < 0.05, compared to HRHF-SL; mean ± SEM shown.

The inflammatory cytokine IL-1alpha and the anti-inflammatory cytokine IL-10 were increased in Flex muscles of HRHF-RL (*p* < 0.05 each) compared to FRC-R and contralateral HRHF-SL (*p* < 0.05 each; Figures 3(a) and 3(b)). However, there were no significant differences in Flex levels of IL-1beta and TNF-alpha between the groups or limbs (Figures 3(c) and 3(d)). Hsp72, an indicator of cell and tissue injury [47, 48], was significantly increased in Flex muscles of HRHF-RL, compared to HRHF-SL and FRC-R (*p* < 0.05, each, Figure 3(e)).

### 3.4. SR Vesicle Ca^2+^ Release and Uptake Rates

**Figure 4:**
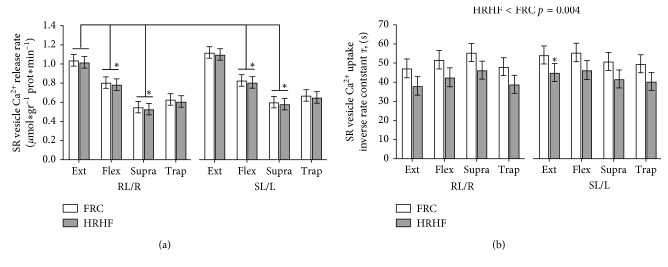
(a) SR vesicle Ca^2+^ release rates for each muscle from each group and limb. The only significant differences were decreased levels in Flex and Supra muscles of each limb, compared to the Ext muscles of HRHF-RL and FRC-R limbs (^*∗*^*p* < 0.05). (b) SR vesicle Ca^2+^ uptake rates, shown as the inverse rate constant (Tau) for muscle. HRHF rats showed higher rates (slower inverse rates) than FRC rats, overall (*p*=0.004), particularly in the Ext muscle of the HRHF-SL limbs, compared to the FRC-L limbs (^*∗*^*p* < 0.05). ANOVA results are shown in individual panels as mean ± SEM.

In all SR vesicle measured in the FRC rats, there were no differences between the right and left limbs for each muscle examined. Therefore, we compared FRC-R limb results to HRHF-RL results and FRC-L to HRHF-SL results. Further, there were no differences between muscle regions A versus B for all analyses, and we therefore presented data from region B only. The SR vesicle Ca^2+^ release rates were similar in the individual muscles of HRHF versus FRC rats (Figure 4(a)). However, there were muscle-specific differences independent of the groups, with overall significantly lower release rates in all Flex muscles (RL/R/SL/L) and Supra muscles (RL/R/SL/L), compared to the Ext muscles (RL/R) (*p* < 0.05 each; Figure 4(a)). Overall, the SR vesicle Ca^2+^ uptake rates were significantly different between the HRHF and FRC rats with an average *τ* difference of −9.2 ± 3.2 seconds adjusted for muscle type (*p*=0.004; Figure 4(b)). The increased SR Ca^2+^ uptake rate in HRHF was consistent in all muscles analyzed. Post hoc analyses revealed that the SR vesicle Ca^2+^ uptake rate was faster in HRHF-SL-Ext (*p* < 0.05), compared to FRC-L-Ext muscles. In summary, 6 weeks of muscle overuse increased the SR Ca^2+^ uptake rate, while the release rate was unaffected by overuse.

### 3.5. Expression of Proteins Involved in the Intramuscular Ca^2+^ Homeostasis

**Figure 5:**
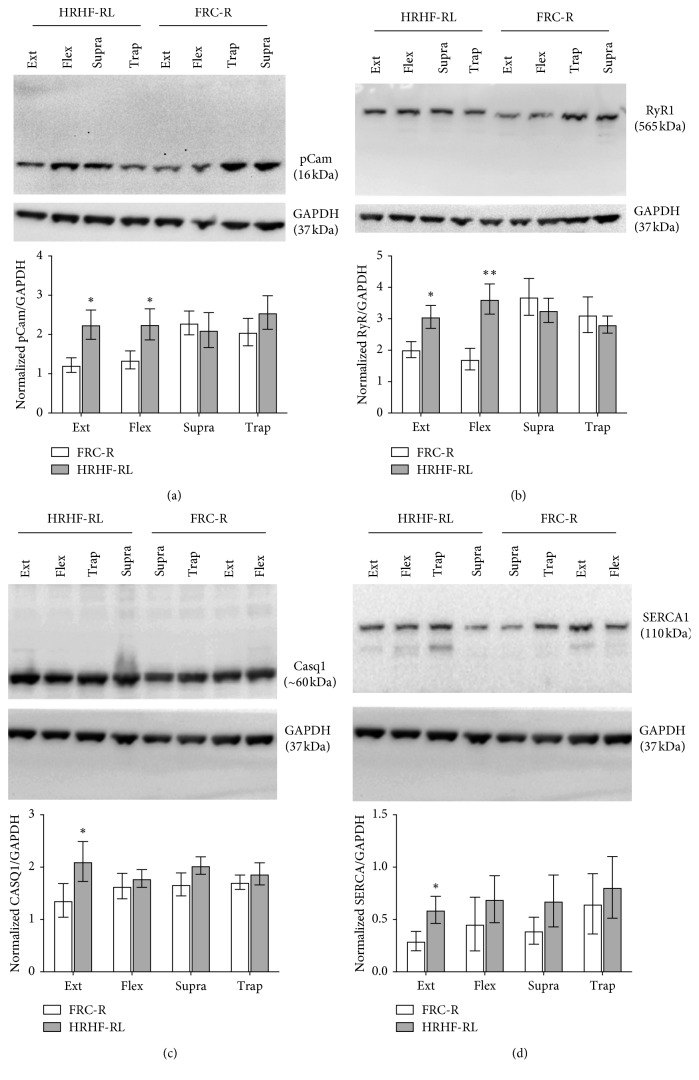
Western blot analysis of muscles. Muscle samples from each limb and muscle subgroup of each rat were analyzed using SDS-Page and western blot analysis (each replicated at least 5 times). Full-length blots are shown for the proteins of interest (pCam, RyR1, Casq1, and SERCA1). Membranes were cut into upper and lower halves and one-half stained for GAPDH (the loading control), and the other for the protein of interest, as shown clearly in (c) and (d). Upper half of the membrane shown in panel C was stripped and reprobed for SERCA (panel D). (a) Representative western blot showing pCam and GAPDH stained bands. pCam showed significantly increased expression levels in the Ext and Flex muscles of HRHF-RL, compared to FRC-R limbs. (b) RyR significantly increased the expression levels in the Ext and Flex muscles of HRHF-RL, compared to FRC-R limbs. (c) Casq1 differed significantly with increased expression levels in the Ext muscles of HRHF-RL, compared to FRC-R limbs. (d) SERCA1 differed significantly with increased expression levels in the Ext muscles of HRHF-RL, compared to FRC-R limbs. ^*∗*^*p* < 0.05 and ^*∗∗*^*p* < 0.01, compared to FRC-R. The order of muscle loaded into individual wells of gels differed across analytes assayed; however, the order is consistently presented in the graphs. ANOVA results are shown in individual panels as mean ± SEM.

The pCam showed significant group differences (*p*=0.03), with post hoc analyses revealing higher levels in the HRHF-RL-Ext and HRHF-RL-Flex muscles compared to the same muscles in FRC-R (*p* < 0.05 each; Figure 5(a)). RyR1 levels showed a significant interaction between the groups and muscles examined (*p*=0.026), with higher levels in HRHF-RL-Ext and HRHF-RL-Flex muscles compared to the same muscles in FRC-R (*p* < 0.05 and *p* < 0.01, respectively; Figure 5(b)). Casq1 levels showed a group difference (*p*=0.049), with higher levels in the HRHF-RL-Ext muscles compared to the same muscle in FRC-R (*p* < 0.05; Figure 5(c)), as did SERCA1 which had higher levels in the HRHF-RL-Ext muscles compared to the same muscle in FRC-R (*p* < 0.05; Figure 5(d)).

## 4. Discussion

**Figure 6:**
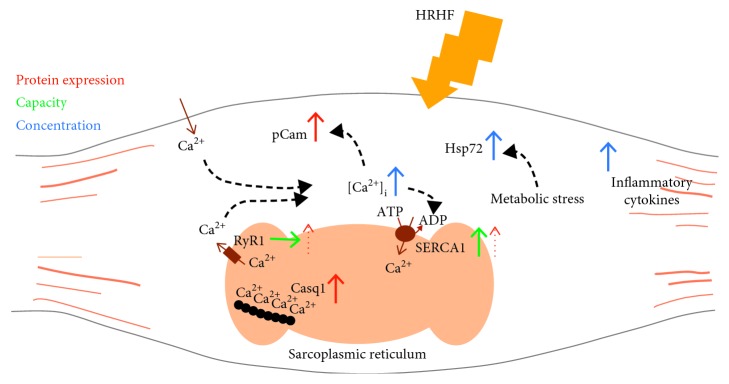
A model for possible early and late events in muscle response to HRHF. Exposure to the HRHF task induces an impaired muscle Ca^2+^ homeostasis leading to an increase in [Ca^2+^]_i_ as indicated by an increase in pCaM. The increased [Ca^2+^]_i_ may be mediated by leaky RyR1 and/or uptake of extracellular Ca^2+^. Further, HRHF induces muscle metabolic stress indicated by an increase in muscle Hsp72. The increased [Ca^2+^]_i_ is accompanied by an increased SR vesicle Ca^2+^ uptake rate and SERCA1 together with increased Casq1 which may lead to an enhanced SR Ca^2+^ buffer capacity. The model points to an altered myocellular Ca^2+^ handling as an early adaptation to muscle overload.

The novel finding of this study is the increased SR vesicle Ca^2+^ uptake rate accompanied by increases in [Ca^2+^]_i_ as indicated by an increase in pCam and protein levels of RyR1 and Casq1 following a exposure of 6 weeks to the HRHF task concomitant with behavioral indices of fatigue and discomfort. This strongly indicates an altered myocellular Ca^2+^ regulation, SR Ca^2+^ handling, and SR protein expression by muscle overload as modelled in Figure 6.

### 4.1. Ca^2+^ Regulation

Our hypothesis of a persistent increase in [Ca^2+^]_i_ in the muscle subjected to HRHF was supported by pCam which was found to be elevated in the HRHF-RL-Ext and HRHF-RL-Flex muscles compared to the same muscles in FRC-R limbs. Exercise has been shown to increase [Ca^2+^]_i_ and pCam in an intensity-dependent manner [49, 50]. The phosphorylation is central in adaptive hypertrophy and metabolic remodeling responses in skeletal muscle [51, 52]. Thus, in response to repeated muscle contraction, elevation in [Ca^2+^]_i_ is well regulated and presents an adaptation to muscle overload rather than a pathological condition. However, a persistently elevated [Ca^2+^]_i_ may lead to degradation of Ca^2+^ regulatory proteins and disruption of proper homeostasis [13, 16, 53]. If [Ca^2+^]_i_ remains elevated for prolonged durations, Ca^2+^ can be transported into the mitochondria to trigger multiple programmed cell death pathways and apoptosis [54].

Sessions of exercise result in alterations in SR Ca^2+^ cycling properties, i.e., decreases in both SR vesicle Ca^2+^ uptake and release rates [10, 11, 55]. Just six weeks of high intensity training can induce an enhanced SR Ca^2+^ release rate due to an enhanced total volume of SR [12].

The SR Ca^2+^ release rate was not increased with HRHF. However, in contrast to findings in earlier studies [56, 57], the SR Ca^2+^ release channel protein RyR was significantly increased, indicating that these channels are not fully functional. The observed [Ca^2+^]_i_ overload may thus be caused by leakage from the SR and/or increased cell membrane influx further activating intracellular Ca^2+^-dependent proteins, such as calpain, that degrade intracellular proteins, cellular membranes, and nuclear DNA [13, 16].

The obtained significant differences in SR vesicle Ca^2+^ release rate between muscle groups could not be explained by differences in fiber type composition, as they have been reported as quite similar in the four muscles studied [58]. In contrast to the release rate, the SR vesicle Ca^2+^ uptake rate was significantly increased with HRHF (*p*=0.004), while SERCA1 protein content only tended to be increased by HRHF (*p*=0.25). Further, there was a clear increase in Casq1, considered as the principal Ca^2+^ binding protein in the SR in Type II fibers. Thus, both the key SR Ca^2+^ binding and pump proteins were increased, clearly indicating an HRHF-related increased Ca^2+^ handling capacity.

The impaired muscle Ca^2+^ homeostasis in all muscles examined was accompanied by adaptations in Ca^2+^ regulation shown as a faster uptake rate of [Ca^2+^]_i_ in HRHF rats compared to FRC rats. The increased SR Ca^2+^ uptake rate may reflect an early adaptation to avoid a detrimental increase in [Ca^2+^]_i_ leading to a sustained increased cytosolic [Ca^2+^]_i_ above normal, similar to previous findings in fatigued muscle [57]. In skeletal muscle, the release and reuptake of Ca^2+^ by the SR govern a crucial role in maintaining [Ca^2+^]_i_, and hence the SR properties are major determinants of muscle function and performance. Still, Ca^2+^ can leak out of the SR either through the RyRs or through the SERCA, under, e.g., metabolic stress or strenuous activity [14]. Any Ca^2+^ leaking out of the SR by any pathway without a balanced reuptake would ultimately be expected to affect the overall muscle function and thus leading to muscle damage.

The present study addresses the detection of early driving mechanisms aiming at maintaining homeostasis but which eventually may be leading to tissue damage. The altered Ca^2+^ homeostasis within the muscle is in line with previously described changes in studies examining mechanisms of a leaking SR.

### 4.2. Indicators of Muscle-Impaired Function and Tissue Injury

Six weeks of exposure to the HRHF task lead to behavioral indices of fatigue and discomfort. Despite likely learning induced increases in mean reach rate, rats showed indices of muscle fatigue in the third session per day in week 3 and in both the 2^nd^ and 3^rd^ sessions per day by week 6, as indicated by reduced reach rate and mean reach impulse (grasp force × grasp duration) [39]. HRHF rats showed several indices of increased muscle discomfort, particularly in the HRHF-RL. Specifically, indices of forelimb muscle discomfort (reduced grip strength and forearm agility, mechanical withdrawal threshold, and increased incidence of supinated rather than pronated pulling, i.e., increased lever pulling with the flexor mass rather than the combined contractions of both flexor and extensor muscles). These findings of early stage sensorimotor declines are in line with previous results using this model [25, 34, 37, 43, 59]. Indicators of pain and fatigue were not as pronounced in the present experimental setup as in previous studies using the same principal model for longer time periods, such as after 18 weeks of HRHF performance when significant reflexive grip strength declines were evident and there was a significant increase in rats sitting in the corner pulling on the lever bar rather than participating (declines not observed here) [25].

We observed a significant increase in IL-1alpha in the HRHF-RL flexor muscles, an inflammatory cytokine that increases in response to muscle injury [60]. The concomitant increase in IL-10, a potent anti-inflammatory cytokine, in the HRHF-RL flexor muscles, suggests that an anti-inflammatory response has been launched. The latter finding is consistent with the increased inducible Hsp72 in the same muscles, proteins thought to be provoked by infiltrating inflammatory cells and anti-inflammatory in nature [61–63]. In mammalian cells, Hsp72 increases after most types of tissue injury and is thought to play key roles in skeletal muscle repair, regeneration, or adaptation [62, 64, 65].

### 4.3. Implications for Work-Related Muscle Disorders in Humans

Early-phase muscle adaptation to repetitive work, with altered Ca^2+^ homeostasis, was evidenced by an increased Ca^2+^ uptake rate in an attempt to keep up with the continued increase in [Ca^2+^]_i._ These disturbances may mirror intracellular changes in the early stage of musculoskeletal disorder development in the human myalgic muscle that may deteriorate the Ca^2+^ regulation and impair muscle function.

The disturbed Ca^2+^ homeostasis on a cellular basis may be due to a constant activation of the same muscle fibers [66]. A subsequent metabolic overload with a higher reliance on anaerobic processes has been indicated by increased levels of lactate and pyruvate in the myalgic muscle [67]. However, in that same study, there was no indication of a higher level of interstitial lactate dehydrogenase that might have indicated membrane leakage in the myalgic muscles [68].

In humans with myalgia, a large number of cytokines and other inflammatory markers have been investigated in serum and tissue biopsies [69]. Increased interstitial concentrations of inflammatory mediators, such as bradykinin and kallidin, have been found in patients with chronic severe trapezius myalgia [70]. However, the early stage of work-related musculoskeletal disorders induced in the present rat model may be more comparable in disease severity to studies on workers with mild myalgia who are studied during the workday. In such populations with less severe disorders, one study observed no indication of increased IL6, in spite of other indications of disturbed metabolism, and a second study observed no increased levels for any of the 10 included cytokines [68, 71].

Regarding Hsp72 in the present rat study, we found a significant increase in Flex muscles of the HRHF-RL compared to the HRHF-SL and FRC-R, while in a human study, we did not find resting baseline differences in Hsp72 among workers with trapezius muscle myalgia compared to healthy controls. However, among the workers with trapezius muscle myalgia, Hsp72 increased ∼8 fold following a 7-hour workday with standardized repetitive work [27]. Interestingly, however, it was shown that Hsp72 decreased following a 10-week strength training period that in previous papers was reported to relieve trapezius muscle pain and improved muscle morphological and metabolic markers [72–74]. Measures of task performance demonstrated a relatively higher muscle load and faster fatigue development in workers with myalgia compared to healthy controls [73]. This clearly shows that heat shock proteins play a focal role in the development of human work-related muscle pain and impaired function as also studied in our rat model. Thus, it is likely that also the underlying Ca^2+^ regulation is instrumental in humans as shown for rats in the present study. Interestingly, recently, dysfunctional regulation of proper Ca^2+^ homeostasis in skeletal muscle has been observed in women with work-related myalgia [18, 75] and in patients with sporadic inclusion body myositis [16].

Work-related musculoskeletal disorders have been considered more of central than peripheral origin; as a consequence, treatments have not been focused on peripheral consequences of mechanical work exposure. Earlier findings of increases in nocioceptive substances, such as glutamate and lactate, in the myalgic muscle [67], substantially support a peripheral component in work-related muscle pain. Such evidence of relationships between physical work exposures versus muscle function and pain, together with explanatory underlying mechanisms of intramuscular subcellular responses, is crucial in the development of effective treatment and prevention of musculoskeletal disorders.

## 5. Conclusion

The study offers an insight into the early events in muscle response to HRHF tasks. Such exposure induces an impaired muscle Ca^2+^ homeostasis seen as an increase in pCam indicating an increase in [Ca^2+^]_i_. This may be mediated by leaky RyR1 and/or uptake of extracellular Ca^2+^. Further, HRHF induces muscle metabolic stress as indicated by an increase in muscle Hsp72. The increased [Ca^2+^]_i_ is accompanied by an increased SR vesicle Ca^2+^ uptake rate together with an increase in Casq1, each leading to an enhanced SR Ca^2+^ buffering capacity. Figure 6 offers an overview of the possible interplay of the muscle responses that may lead to early-phase muscle overload and pain.

Previous results from human studies indicate that the proposed model of the Ca^2+^ regulation interplay may be instrumental in early development of musculoskeletal disorders in human myalgic muscles. Implications for human work-related musculoskeletal disorders from this rat model include the importance of mechanical work exposure and the clear indication of a peripheral contribution to the pathomechanisms. Both aspects must be considered when designing effective treatment and prevention of musculoskeletal disorders.

## Figures and Tables

**Table 1:** Overview of analyses performed per group, limb, and type of analysis.

Rat group	Limb	Muscles	Ca++ uptake/release	Western blot	ELISA	Task exposure calculations	Sensorimotor behavioral tests
HRHF	Reach limb, RL					X	X
		M. flexor digitorum, Flex	X	X	X		
		M. extensor digitorum, Ext	X	X			
		M. trapezius, Trap	X	X	X		
		M. supraspinatus, Supra	X	X			
HRHF	Support limb, SL					X	X
		M. flexor digitorum, Flex	X		X		
		M. extensor digitorum, Ext	X				
		M. trapezius, Trap	X				
		M. supraspinatus, Supra	X				
FRC	Right limb, R					N/A	X
		M. flexor digitorum, Flex	X	X	X		
		M. extensor digitorum, Ext	X	X			
		M. trapezius, Trap	X	X	X		
		M. supraspinatus, Supra	X	X			
FRC	Left limb, L					N/A	X
		M. flexor digitorum, Flex	X		X		
		M. extensor digitorum, Ext	X				
		M. trapezius, Trap	X				
		M. supraspinatus, Supra	X				

HRHF = high repetition high force rats, *n* = 6; FRC = food-restricted control rats, *n* = 6; N/A = not applicable.

## Data Availability

The data used to support the findings of this study are available from the corresponding author upon request.

## Supplementary Materials

Supplementary Materials Supplementary Figure 1: body weights across the course of the experiment.
