# Supplementary figures and images for: Calcium Fluxes in Work-Related Muscle Disorder: Implications from a Rat Model
Source: Biomed Res Int. 2019 Sep 30;2019:5040818. doi: 10.1155/2019/5040818 (PMC6791278; doi:10.1155/2019/5040818)

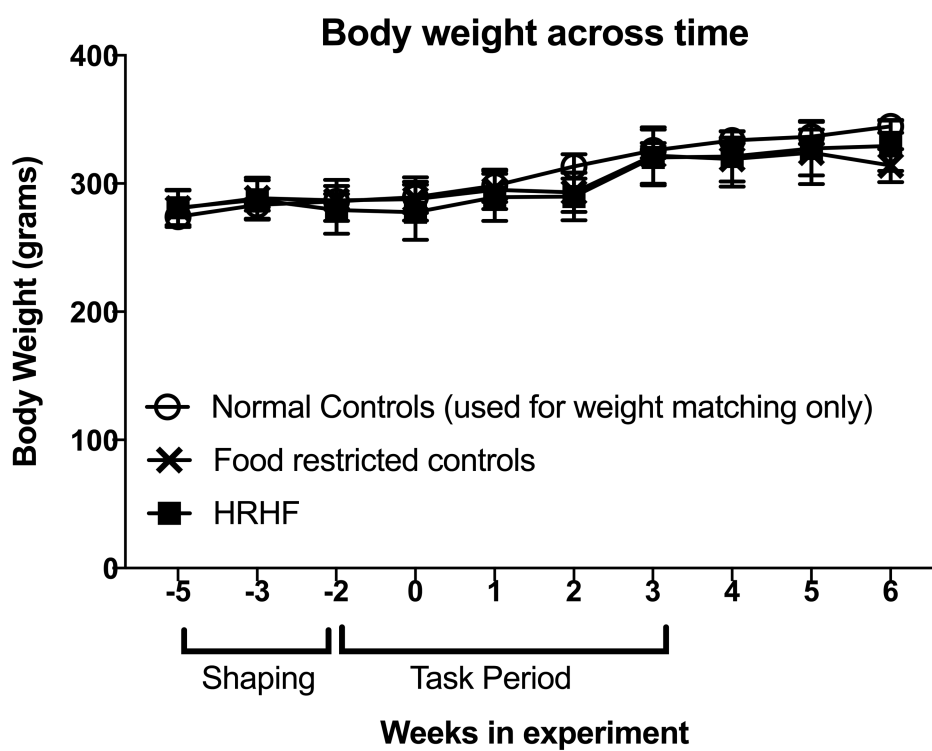

Supplement: Supplementary Materials — Supplementary Figure 1: body weights across the course of the experiment. [file 5040818.f1.pdf]
